# Relationship between mental workload and musculoskeletal disorders with intention to leave service among nurses working at neonatal and pediatric departments: a cross-sectional study in Iran

**DOI:** 10.1186/s12912-024-02112-7

**Published:** 2024-06-26

**Authors:** Elham Naserian, Shahnaz Pouladi, Razieh Bagherzadeh, Maryam Ravanipour

**Affiliations:** 1 Student Research Committee, Department of Nursing, School of Nursing and Midwifery, Bushehr University of Medical Sciences, Bushehr, Iran; 2 Department of Nursing, School of Nursing and Midwifery, Bushehr University of Medical Sciences, Bushehr, Iran; 3 Department of Midwifery, School of Nursing and Midwifery, Bushehr University of Medical Sciences, Bushehr, Iran; 4 The Persian Gulf Tropical Medicine Research Center, The Persian Gulf Biomedical Sciences Research Institute, Bushehr University of Medical Sciences; Department of Nursing, School of Nursing and Midwifery, Bushehr University of Medical Sciences, Rishehr Street, P.O. Box: 7518759577, Bushehr, Iran

**Keywords:** Intention, Leave the service, Iran, Mental workload, Musculoskeletal disorders, Neonatal nurses, Pediatric nurses, Shift work schedule

## Abstract

**Background:**

Despite the challenge of nursing shortage in the world and its subsequent impact on care quality as well as aggravation of the situation by intention to leave service, this issue has not been properly addressed, especially among neonatal and pediatric nurses. The present study aims to identify the relationship between mental workload and musculoskeletal disorders with intention to leave the service among nurses working at neonatal and pediatric departments.

**Methods:**

This descriptive-analytical study was conducted on 145 nurses working at neonatal and pediatric departments in six hospitals in Bushehr Province using full-census method. The data were collected using national aeronautics and space administration-task load index (NASA-TLX), Cornell musculoskeletal discomfort questionnaire(CMDQ) and Mobley and Horner’s voluntary turnover questionnaire. The data were analyzed using descriptive statistics, independent t-test, Mann-Whitney U test, one-way analysis of variance (ANOVA), Kruskal-Wallis test, Pearson’s and Spearman correlation tests and hierarchical linear regression in simultaneous model in SPSS 19.0.

**Results:**

The mean score of intention to leave the service was 9.57 ± 3.20 (higher than the moderate level) and the mean mental workload was 71.65 ± 15.14 (high level). Pain in at least one of the legs (100%), back (77.3%) and knees (76.6%) was highly prevalent. However, no statistically significant correlation was found between musculoskeletal disorder categories and intention to leave the service (*p* > 0.05). The regression analysis results revealed among mental workload domains, only effort-induced workload was negatively and significantly correlated with intention to leave the service (*p* = 0.003; β=-0.078). However, the number of night shifts per month was positively and significantly correlated with intention to leave the service (*p* = 0.001; β = 0.176).

**Conclusions:**

Planning for appropriate allocation of night shifts, investigating the etiology of musculoskeletal disorders and providing solutions for reducing mental workload should be prioritized by policymakers, while maintaining pediatric nurses’ motivation for making efforts.

## Background

Leaving the service refers to the attrition of an organization’s workforce during a certain period of time [1]. Intention to leave the service is a cognitive stage that occurs before the actual turnover and is defined as the mental decision-making about leaving or staying in the service [2]. Nurses’ intention to leave the service has been reported 3–75% at the international level [3]. One study reported nurses’ intention to leave the service as 32.7% in Iran. In another study, this rate was 54.6% [4, 5]. Leaving the service is among the major reasons for lack of human resources in the health system, which has become a challenging and international problem [6]. The organization for economic cooperation and development (OECD) reported more than 3.2 million nurses will be needed by 2030 [7]. Leaving the service causes the loss of efficient and competent nurses [8], affecting the quality of care and services provided to patients [9]. Investigating the etiology of this phenomenon is complex, especially in healthcare settings, because it could be influenced by motivational, cognitive and behavioral factors. Moreover, the factors associated with nurses’ intention to leave service could be divided into two categories, i.e., individual and organizational factors [10]. Previous studies have identified various factors influencing nurses’ intention to leave the service. In short, good salary, good relationship with the manager and appropriate benefits were associated with less intention to leave the service, while excessive absenteeism, work stress, burnout, service dissatisfaction and workload were associated with higher nurses’ intention to leave the service [11, 12]. Among the above-mentioned factors, service dissatisfaction was the most important one, which was associated with service stress, shortage of nursing staff, increased patient-to-nurse ratio, burnout and nurses’ high workload [13]. Workload is a general concept, which includes both physical and mental workload, analyzing which separately could provide different results regarding the intention to leave the service. The physical workload of nurses is defined as physical skills such as moving and transferring patients and administering medicine [14]. Mental workload is a multidimensional and multifaceted concept, which is defined as the effort made by the mind while performing tasks. In other words, mental workload refers to the intellectual and cognitive requirements or analytical efforts made by employees while performing tasks at a specific time and place [15]. Mental workload includes receiving, understanding and interpreting information, making decisions, concentrating and interacting with patients and their families [16]. Studies have reported mental workload as 70.21% and 56.4% among Indonesian and Chinese nurses, respectively [17, 18]. The mental workload of emergency nurses and nurses caring for patients with COVID-19 has been reported as 85.42% and 67.14%, respectively, in Iran [19, 20]. Increased mental workload is associated with workforce’s fatigue and injury, which could affect nurses’ clinical performance [20]. Moreover, mental workload is among the psycho-social factors that could affect physical aspects such as work-related musculoskeletal disorders [21, 22]. Musculoskeletal disorders are associated with psychological stress as well as physical workload [23]. Work-related disorder is among the categories of musculoskeletal disorders, which has been investigated in different work environments such as administrative, manufacturing and healthcare settings [24–26]. The prevalence of musculoskeletal disorders has been reported as 48.1–95.7% among different societies [27–29]. This disorder is prevalent among healthcare staff and is more prevalent among nurses and physiotherapists [23, 30]. Musculoskeletal disorders are highly prevalent among Iranian nurses [31, 32], especially in the back, neck, upper back, knees, ankles, hands, shoulders, hips, thighs and elbows [33]. Furthermore, the nature of nurses’ profession exposes them to fatigue and diseases, especially musculoskeletal disorders [34]. The nursing profession consists of different subgroups, in each of which nurses perform different tasks under different working conditions. Pediatric nursing is one of these subgroups, in which nurses provide appropriate and quality care for children in acute and chronic care units [35]. Although working in other wards such as emergency room/ward is more associated with physical and musculoskeletal disorders, it seems necessary to examine working conditions in pediatric wards because pediatric nursing actions like blood sampling, catheterization, tracheal intubation, etc. are often performed under inappropriate conditions [36]. Poor cooperation with nurses in providing care due to parents’ excessive concern about their sick children [37], lack of agreement on pain management and necessity of assisting patients with activities like eating, dressing and bathing make taking care of children more challenging than taking care of adults [38]. Musculoskeletal disorders have acute and chronic physical consequences, which often affect other aspects of health and well-being. Some functional consequences could be easily identified such as taking analgesics to cope with symptoms, while others affect life quality at work and home, and are more likely to reduce or change work tasks and recreation [39, 40]. In addition to physical factors, it has been reported that pediatric nurses have high mental workload [37]. Most of nurses working at pediatric intensive care units have reported temporal demand, cognitive load and psychological stress as the major workload components [41]. Moreover, working at crowded clinics with children in different age groups on a tight schedule could also affect pediatric nurses’ intention to leave the service [42]. In addition to taking care of sick children, pediatric nurses also take care of their family members in some way. In Iran, the majority of pediatric nurses are women who become strongly emotional due to their sense of motherhood when caring for critically ill children and observing their parents [43]. Nurses feel distressed when performing painful procedures on children and describe it even as “torture” for their patients [44]. When caring for adults, communication is often established between the medical team and patients, so that patients could directly involve in treatment and declare their agreement/disagreement with the treatment process. However, when taking care of children, communication is established between the child, parents and medical team. In some cases, parents are more involved in choosing treatment strategies due to the prevalence of patriarchal culture in Iran and children’s preferences are less considered. Furthermore, conflicts between children and parents’ and caregivers’ demands may cause moral tensions in providing child care [45]. Being precise and sensitive and having high concentration to provide nursing care to children and infants are of particular importance, so that any negligence could lead to irreparable consequences. Facing such stresses and tensions, the need to go back and forth to the patient’s bed and frequently visit the sick child, and nature of providing nursing care to children for a long period of time, which often causes them to be in improper ergonomic positions, may affect nurses’ mental workload, physical health and intention to leave the service. However, a limited number of studies have been conducted in this filed. Consequently, since it is necessary to perceive the intention to leave the service and potential effective factors for planning, policy making and performing appropriate interventions in order to maintain specialized human resources, this research was conducted to determine the relationship between the mental workload and musculoskeletal disorders and intention to leave the service among nurses working at neonatal and pediatric departments in Bushehr Province, during 2022 to 2023.

## Methods

This cross-sectional descriptive, analytical research was conducted in Bushehr Province from December 2022 to April 2023. The research environment included all hospitals affiliated to Bushehr University of Medical Sciences and the research community consisted of nurses working at neonatal and pediatric departments. The inclusion criteria were having a diploma in auxiliary nursing or a university degree in nursing or midwifery, having informed consent to participate in the research and having at least one year of full-time work experience at neonatal or pediatric departments. The exclusion criteria were not completing the questionnaires, having a history of any musculoskeletal disorder such as fracture, joint dislocation, ligament strain, etc. as well as pregnancy and menopause. Considering that hierarchical linear regression analysis was used to confirm or reject the research hypotheses and according to the sample size rule for regression analysis, i.e., choosing 10–30 samples for each predictor (*n* = 10-30k), 10 samples were considered for each predictor variable. Due to having two main predictor variables, all the main demographic variables were considered as the potential predictors. Finally, 130 samples were considered due to having 13 predictor variables. However, the sample size was determined to be 143 nurses, taking into account the withdrawal rate of 10%. Given that the research community included 204 individuals, full-census sampling method was used, so that a questionnaire was given to all 171 individuals who met the inclusion criteria and, finally, 145 complete questionnaires were analyzed (response rate: 84%). It is noteworthy that this study was a master’s thesis and the researchers did not have any power relations with the participants or the coordinators of the permission to enter the environment.

Demographic questionnaire included information such as age, marital status, educational level, body mass index, history of vitamin D deficiency (self-report), having children, age of the youngest child, hospital and department name, employment status, nursing experience, number of night shifts, monthly overtime hours and type of work shifts.

National aeronautics and space administration-task load index (NASA-TLX): This questionnaire is a multi-dimensional assessment tool, which evaluates and measures the perceived mental workload in order to assess a specific task or activity. NASA-TLX consists of subscales of mental demand, temporal demand, physical demand, performance, effort and frustration.

These subscales are explained as follows:

Mental workload(demand): How much mental and cognitive activity was required? Was this task easy or difficult, simple or complex?

Physical workload(demand): How much physical activity was required? Was this task easy or difficult? Was it followed by rest or did it require intense physical activity?

Temporal demand: How much time burden did you feel based on the speed of performing each activity? Did the activity progress quickly or slowly?

Performance: How successful were you in performing the goals of the task? How satisfied were you with your performance?

Frustration level: How much did you feel discouraged, irritated and stressed during work, instead of feeling secure, relaxed and satisfied?

Effort: How hard did you have to work mentally or physically to reach the desired performance?

This tool includes two parts: In the first part, each subscale is scored from 0 to 100 based on a 5-point scale. Each subscale is presented as a 10 cm line with a title, e.g., temporal demand. The top point is expressed by a bipolar descriptor (high/low). Among the six NASA-TLX subscales, three subscales contribute to the needs imposed on the nurse when performing physical, mental and temporal tasks, and three subscales are related to the result of performing the task, i.e., personal performance, level of effort and level of failure and frustration. Except for the performance subscale which is assessed based on good and bad options, other subscales are evaluated based on low and high options. Explanations are provided for each of these six subscales, which should be read by users before scoring. In the second part, a pair-wise comparison is made between these subscales in 15 modes, and the participant specifies the subscale that has the most impact on them. The total mental workload score is obtained by multiplying the score of each subscale in the first stage by the corresponding weight in the second stage (number of times each subscale is chosen), which is then divided by 15. The scores below 50 are considered acceptable and scores above 50 are considered high [46, 47]. Mohammadi et al. (2013) confirmed the face and content validity of this tool. Its internal consistency reliability was obtained as 0.84 using Cronbach’s alpha method, indicating appropriate reliability [48].

Cornell musculoskeletal discomfort questionnaire (CMDQ): This questionnaire was organized in three parts: Discomfort frequency, severity of pain and effect on work power during the last working week [43, 44]. CMDQ consists of a map depicting 12 body parts (in total, 20 regions of the body). This questionnaire is designed in standing and sitting positions both for men and women. The standing questionnaire has been localized and includes three parts of discomfort frequency, severity of pain and effect of pain on work power. Scores could be analyzed by weighting the scores to identify the most serious problems. Discomfort frequency is first specified by scoring never (0), 1–2 times a week (1.5), 3–4 times a week (3.5), every day (5) or several times a day (10). Then, weighting is done by multiplying the above-mentioned score (0, 1.5, 3.5, 5, 10) by discomfort (1, 2, 3) and interference (1, 2, 3) scores. Thus, the score of each part of the body ranges from 0 to 90. Based on CMDQ scoring, musculoskeletal disorders are divided into three general categories: Trunk, upper and lower limbs. The questionnaire’s validity and reliability were confirmed by Oğuzhan Erdinç et al. (2008). The reliability of frequency, severity and interference was obtained as 0.876, 0.895 and 0.875 using the test-retest method, respectively, indicating high CMDQ internal consistency [49]. Kashani et al. (2009) confirmed face and content validity of this tool, and its reliability was obtained as 0.98 using Cronbach’s alpha method. Cronbach’s alpha coefficients of discomfort frequency, severity of pain and effect of pain on work power were obtained as 0.95, 0.96 and 0.96, respectively [50].

Voluntary turnover intention scale: This scale was designed by Mobley and Horner (1978) based on a five-point Likert scale, so that the items are scored from 1 (completely disagree) to 5 (completely agree). This scale consists of three items. The lowest and highest scores are 3 and 15, respectively. The construct validity was assessed by Mobley et al. (1978) and the results showed the scale has adequate construct validity. The scale Cronbach’s alpha was reported as 0.90 [51]. Abid et al. (2017) in Pakistan calculated reliability as 0.86 using Cronbach’s alpha [52]. Also, the scale validity and reliability were confirmed by Hashemi et al., in Iran so that the reliability was reported as 0.89 [53].

### Data collection

After obtaining the Code of Ethics and permission to enter the research setting included six university hospitals in different cities of Bushehr Province, the staff list was prepared through nursing offices and visited neonatal and pediatric departments in different work shifts on several days after coordinating with supervisors and head nurses. The necessary explanations were given to nurses regarding the research objectives and methodology and they were invited to participate in the study. The research tool was available in online and printed versions, which was used based on the participants’ preference. If the participants wish to complete the questionnaires online, the informed consent form link and questionnaires were provided to them via short messages (SMS) and, finally, their responses were recorded. The printed questionnaires were collected by the researcher after being completed by the nurses.

### Data analysis

The data were analyzed using SPSS 19.0 and descriptive statistics, independent t-test, Mann-Whitney U test, one-way analysis of variance (ANOVA), Kruskal-Wallis test, Pearson/Spearman correlation tests and hierarchical linear regression with inter method. The significance level was considered less than 0.05 in all cases.

### Ethical considerations

This research was approved by Ethics Committee of Research Assistant of Bushehr University of Medical Sciences with reference no. IR.BPUMS.REC.1401.072. After explaining the research objectives and methodology, the informed consent form was obtained from the participants. Moreover, the participants were assured that their information would be kept confidential and they would be informed of the research results.

## Results

Analysis was performed on 145 nurses and midwives. The participants’ demographic characteristics showed all caregivers were female. The participants’ mean age was 32.01 ± 6.31 years old, ranging from 20 to 49 years old. The mean nursing experience was 9.14 ± 6.00 years.

The majority of the nurses participating in the research were married (80.7%) and their educational level was bachelor’s degree or higher (78.60%) (Table 1).

**Table 1:** The demographic characteristics and job-related variables of the participants (*n* = 145)

Variable		Mean* or Frequency	Standard deviation** or percent
Age		32.01*	6.31**
Nursing work experience		9.14*	6.00**
Number of night shifts per month		6.52*	3.40**
Monthly overtime		59.65*	44.05**
BMI		25.59*	3.66**
Musculoskeletal discomfort	Trunk	18.24*	20.76**
	Upper limb	5.83*	11.36**
	Lower limb	9.23*	14.67**
Marital status	Single	28	19.3
	Married	117	80.7
Degree of education	Diploma	31	21.4
	Bachelor and above	114	78.6
Self-reported to have vitamin D deficiency	Yes	67	46.2
	No	78	53.8
Having a child	Yes	97	66.9
	No	48	33.1
Age of the youngest child	Under 4 years	30	20.7
	4 to 10 years	41	28.3
	Above 10 years	26	17.9
Department of service	Pediatric	54	37.2
	Neonatal	24	16.6
	Pediatric ER	27	18.6
	PICU	12	8.3
	NICU	28	19.3
Employment status	Types of contracts	14	9.7
	Conscription law’s conscripts	22	15.2
	Semi-permanent employment	21	14.5
	Official	88	60.7
Shift work status	Fixed shift	14	9.7
	Rotating shift	131	90.3

**Table 2:** Comparison mean score of intention to leave service in the levels of qualitative demographic variables in nurses (*n* = 145)

variable	Variable classes	Mean and standard deviation of Intention to Leave service	statistics	significance level
marital status	Single	10.5 $\pm 3.25$	1.730*	0.089
	married	9.34$\pm$3.17		
degree of education	diploma	8.94$\pm$3.18	-1.237*	0.218
	Bachelor and above	9.74$\pm$3.20		
Self-reported to have vitamin D deficiency	Yes	9.42$\pm$3.18	-0.513*	0.609
	No	9.69$\pm$3.24		
having a child	Yes	9.21$\pm$3.12	-1.924*	0.056
	No	10.30$\pm$3.28		
Age of the youngest child	Under 4 years	8.70$\pm$3.51	2.384***	0.072
	4 to 10 years	9.71$\pm$2.39		
	Above 10 years	8.81$\pm$3.49		
Department of service	pediatric	9.85$\pm$3.28	2.157****	0.707
	Neonatal	8.96$\pm$3.58		
	Pediatric ER	9.93$\pm$2.95		
	PICU	9.17$\pm$3.81		
	NICU	9.36$\pm$2.77		
Employment status	Types of contracts	8.93$\pm$2.95	5.608****	0.132
	conscription law’s conscripts	10.82$\pm$4.10		
	Semi-permanent employment	9.10$\pm$3.33		
	Official	9.47$\pm$2.92		
shift work status	fixed shift	9.79$\pm$3.33	-0.232**	0.788
	rotating shift	9.54$\pm$3.20		

* The independent t-test was performed and the reported statistic is T values; **The Mann-Whitney test was performed and the reported statistic is Z values; ***The one-way analysis of variance was performed and the reported statistic is F values; ****The Kruskal-Wallis test was performed and the reported statistic is X2 values

**Fig. 1:**
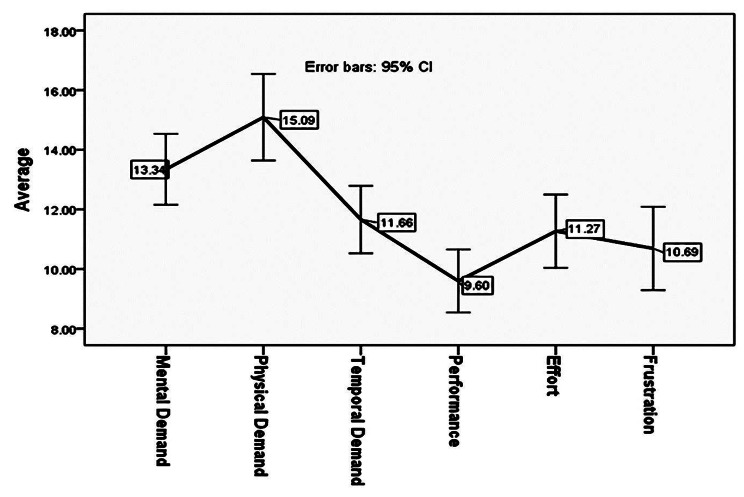
The mean score of different domains of nurses’ mental workload

**Fig. 2:**
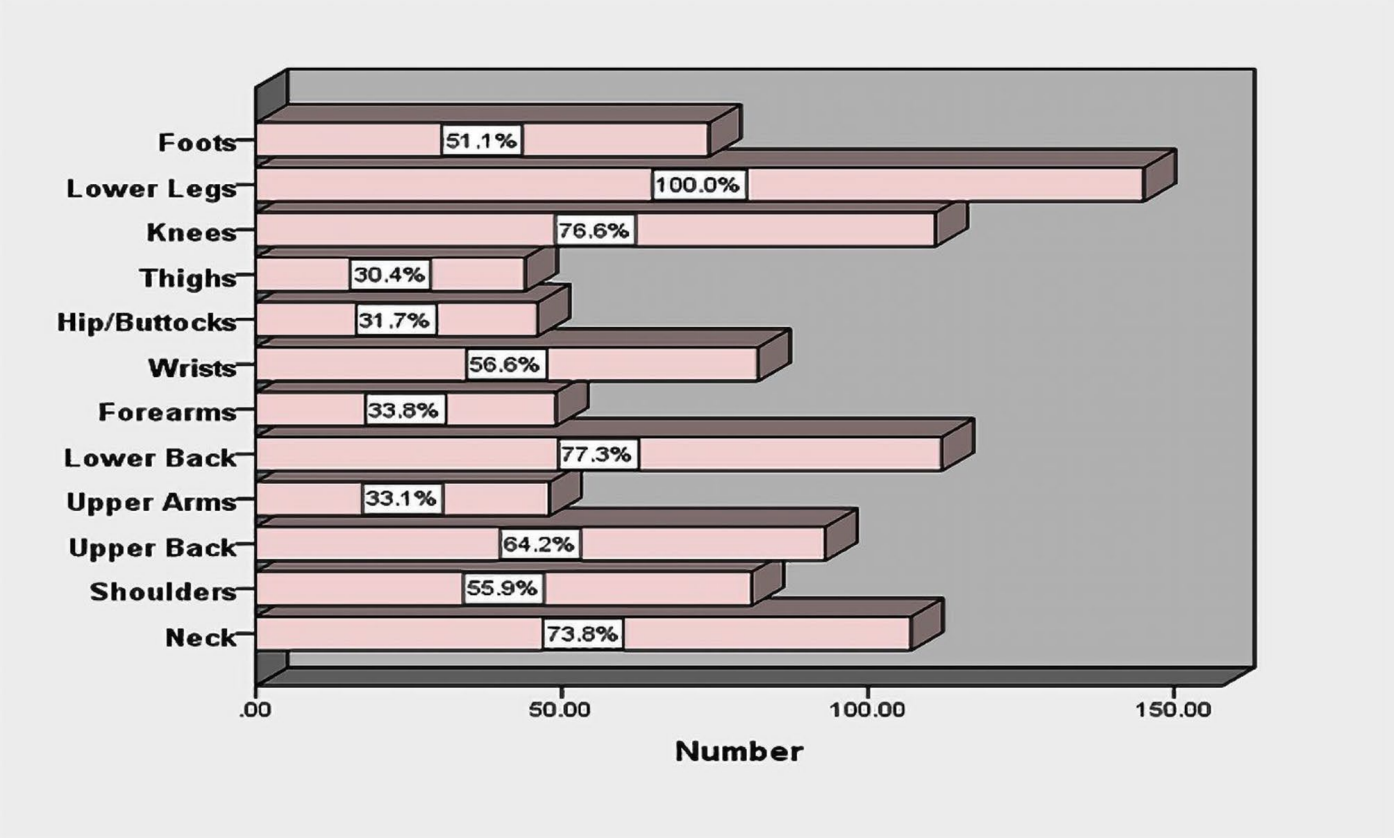
The prevalence of musculoskeletal disorders in participants’ different areas

The mean scores of total mental workload and intention to leave the service were 71.65 ± 15.14 and 9.57 ± 3.20, respectively. The results revealed that the Mean score of intention to leave service in the different levels of qualitative demographic variables in nurses were not statistically significant (*p* > 0.05) (Table 2). Also, Fig. 1 illustrates the mean score of different domains of nurses’ mental workload. Figure 2 shows the prevalence of musculoskeletal disorders in participants’ different areas.

The results revealed none of the categories of musculoskeletal disorders had a statistically significant correlation with intention to leave the service (*p* > 0.05)(Table 3). Also, there was no statistically significant correlation between total mental workload and intention to leave the service (*p* = 0.855). Among the mental workload domains, a negative and significant correlation was found between the burden imposed by the effort (*p* = 0.003) and intention to leave the service. Also, a positive and significant correlation was observed between the mental workload imposed by frustration and intention to leave the service(*p* = 0.028). Among quantitative demographic variables a negative and significant correlation was found between the age and nursing work experience (*p* = 0.003) with intention to leave the service; and a positive and significant correlation was found between number of night shifts per month (*p* = 0.001) and monthly overtime (*p* = 0.020) with intention to leave the service. (Table 3).

**Table 3:** Correlation between quantitative variables with the intention to leave the service (*n* = 145)

Variable		The correlation coefficient	The significance level
Musculoskeletal discomfort	trunk	0.092*	0.269
	Upper limb	0.152*	0.067
	lower limb	0.144*	0.084
Mental workload	Mental Demand	-0.007	0.930
	Physical Demand	0.065	0.436
	Temporal Demand	-0.144*	0.085
	Performance	0.151	0.070
	Effort	-0.244	0.003
	Frustration	0.182	0.028
	total of mental workload	0.015	0.855
Quantitative demographic variables	age	-0.242	0.003
	Nursing work experience	-0.248	0.003
	Number of night shifts per month	0.269*	0.001
	Monthly overtime	0.193	0.020
	body mass index (BMI)	-0.017	0.843

The Pearson’s or Spearman’s correlation test was performed. *The Spearman’s correlation test was performed

Finally, hierarchical linear regression was used to identify the predictive factors of the intention to leave the service. In the first model, only demographic variables such as age, nursing experience, number of night shifts per month and monthly overtime hours were included in the regression. In the second model, two domains of mental workload that were associated with intention to leave the service in previous analyses, i.e., mental workload imposed by the effort and mental workload imposed by frustration, were added to the model. In the first model, demographic variables altogether predicted 10.4% of changes in the intention to leave the service and the model was statistically significant. In the second model that two domains of mental workload were included, the variables altogether explained 14.7% of changes in the intention to leave the service, and the model was statistically significant. In the second model that all the variables were included, the burden imposed by the effort had a negative and significant correlation with the intention to leave the service, i.e., increased score of this domain of mental workload decreased the score of intention to leave the service. There was no statistically significant correlation between the burden imposed by frustration and intention to leave the service. Among the demographic variables, only the number of night shifts per month had a positive and significant correlation with intention to leave the service, i.e., increased number of night shifts increased the intention to leave the service. As a result, based on the regression results of Table 4, in the final model, the mental work load caused by effort had a negative and statistically significant relationship with the intention to leave the service, and the number of night shifts had a positive and statistically significant relationship with the intention to leave the service.

**Table 4:** Regression results to investigate the relationship between mental workload (two areas of workload due to effort and workload due to failure)

Variable		Unstandardized regression coefficient	standard error	Standard regression coefficient	t	significance level	95% confidence interval for the regression coefficient		Statistics and significance level
		B					Low range	High domain	
First model	Constant	9.794	2.294	-	4.270	0.001	5.259	14.329	R square = 0.104 F = 4.047 *P* value = 0.004
	age	-0.033	0.088	-0.064	− 0.371	0.712	-0.206	0.141	
	Nursing work experience/year	-0.061	0.092	-0.114	− 0.658	0.512	-0.243	0.122	
	Number of night shifts per month	0.162	0.089	0.172	1.809	0.073	-0.015	0.338	
	Monthly overtime/hours	0.005	0.007	0.072	0.794	0.428	-0.008	0.018	
Second model	Constant	10.444	2.425	-	4.307	0.001	5.649	15.240	R square = 0.147 F = 3.970 *P* value = 0.001
	age	-0.041	0.088	-0.081	-0.470	0.639	-0.215	0.132	
	Nursing work experience/year	-0.028	0.093	-0.053	-0.303	0.762	-0.211	0.155	
	Number of night shifts per month	0.176	0.088	0.187	1.999	0.048	0.002	0.350	
	Monthly overtime/hours	0.001	0.007	0.018	0.192	0.848	-0.012	0.015	
	Mental load of work caused by effort	-0.078	0.036	-0.183	-2.202	0.029	-0.149	-0.008	
	Mental load of work caused by failure	0.033	0.032	0.089	1.043	0.299	-0.030	0.097	

Dependent variable: intention to leave service

## Discussion

This study was conducted to identify the relationship between mental workload and musculoskeletal disorders and intention to leave the service among nurses working at neonatal and pediatric departments in Bushehr Province.

The results showed pediatric nurses’ intention to leave the service was more than the moderate level (9.57 ± 3.20) and mental workload was at the high level (71.65 ± 15.14), so that among different domains of mental workload, the lowest mean was related to performance and the highest mean was related to physical demand. The results revealed the prevalence of musculoskeletal disorders among nurses was at the unfavorable level, and suffering from pain at least in one leg, neck, back and knees was highly prevalent.

In line with the main research objective, the results showed no correlation between the mean total mental workload and intention to leave the service. Among mental workload domains, only effort, which means performing tasks with diligence, was negatively correlated with intention to leave the service, so that increasing the effort score decreased intention to leave the service. In this regard, Li et al. (2011) conducted a study on nurses in seven European countries and found that intention to leave the service increased by reward frustration such as poor salary and promotion prospects and lack of esteem [54]. However, it is probable that great effort is due to passion for the service, and someone who is interested in their service is not willing to leave it. Carswella et al. found a correlation between nurses’ workload perception and intention to leave the service [55]. Holland et al. showed the perceived workload was correlated with the increased intention to leave the service, and nurses’ satisfaction with work-life balance had a mediating effect on the correlation between the perceived workload and increased intention to leave the service [56].

Furthermore, the results revealed no statistically significant correlation between musculoskeletal disorders and intention to leave the service, which was in line with results of Kox et al. [57]. However, Martinez et al. (2022) conducted a study on Brazilian nurses and reported that conditions leading to pain or musculoskeletal disorders are among the factors influencing the intention to leave the profession [58]. Schug et al. (2022) conducted research in German hospitals and found that infection with COVID-19, pre-existing illness, exhaustion and fear of becoming infected were among the major predictors for sick leave among nurses [59]. Hasselhorn et al. (2005) showed a significant correlation between bending and lifting activities and the intention to leave the profession, and this phenomenon was more common among nurses with a higher level of disability [60].

The results revealed musculoskeletal disorders were highly prevalent among nurses participating in the present study, but it could not affect the intention to leave the service. It seems that although musculoskeletal disorders were highly prevalent and the participants felt pain, they did not feel disabled and continued their work. Moreover, it is probable that personal and family needs have caused them to continue working. Also, it is possible that service satisfaction, especially feeling satisfied with helping others, has caused nurses to continue their work despite workload and suffering from musculoskeletal disorders. The integration of pediatric nurses’ sense of motherhood and their role of providing care could be another reason for lack of correlation between musculoskeletal disorders and intention to leave the service, which could be investigated based on Meyer and Allen’s three-component conceptualization model of organizational commitment. In this model, people stay in their profession because either they are emotionally attached to the organization’s values and goals or they are committed or they know that they will lose a lot if they leave their profession. Therefore, organizational commitment acts as a stabilizer in strengthening employees’ behavioral intentions and plays a significant positive role in retaining nurses [61]. It seems that the reasons that persuade nurses to leave their service are complex and are influenced by individual and organizational factors and may be due to diversity in healthcare centers and categories of health professions. Almost all the nurses of neonatal and pediatric wards participating in the research were female with the mean age of 32.01 ± 6.31 years old and mean work experience of about 10 years, who had higher mean age compared to those working in some wards such as the emergency room where mostly young and male nurses work, and on the other hand, most of nurses participating in this study were married and this point could be the reason why they preferred to maintain their life stability. Also, reasons other than being involved in life issues could be effective in nurses’ intention to leave the service, e.g., financial consequences of leaving the service, especially in the current economic conditions caused by high inflation in Iran and difficulty of finding another service, particularly, for women, could be considered.

so that according to Statistical Center of Iran (2022), the economic female labor force participation rate in the whole country was only about 13.6% of the country’s labor force [62]. It seems that women try their best to keep their services due to social image, financial independence and gender equality that they have experienced as a result of being employed. Therefore, despite high mental workload and discomfort associated with musculoskeletal disorders, pediatric nurses continue their work.

Among demographic and occupational variables, working night shifts was positively correlated with intention to leave the service considering that most of nurses had rotating shifts and evidence has shown working night shifts is associated with mental, physical and family complications [63], it could be analyzed that disrupted sleep-wake cycle is an important consequence of working night shifts. This disorder could negatively affect employees’ sleep quality and quantity, ability to be active and energetic during the day and physical and mental conditions [64]. Saksvik et al. (2011) investigated “individual differences in tolerance to shift work” and showed night shifts could cause fatigue, sleep disturbance and drowsiness [65]. Therefore, the obtained results could be explained in this way that those who have more night shifts generally experience the most time inconsistency with other family members as they feel tired the next day they could not devote enough time to their family roles [66, 67]; and they devote less time to leisure, household tasks, child care and friends and family [68]. Considering these factors, dissatisfaction may arise in the family, which makes these individuals gradually tired of their services, leading to intention to leave the service [69]. It is also possible that most men do not like their wives to have a night shift service, this could be among the reasons for the increased nurses’ intention to leave service. From the organizational viewpoint, the number of nurses in night shifts is less, which could increase the error rate [68], and nurses’ stress, causing fatigue, dissatisfaction [69], and intention to leave service. This could be considered a serious concern as women constitute the majority of the nursing workforce. Therefore, special attention should be paid to the effect of night shift on nurses’ intention to leave service, and appropriate measures should be taken to reduce its complications such as flexibility in planning work shifts, planning rest breaks in staff rest rooms, serving healthy foods in the workplace, sports facilities and providing child care services [68].

To the best of our knowledge, this was the first study conducted on the intention to leave the service among nurses working at neonatal and pediatric departments in Iran, which was of particular importance due to the importance of retaining specialized nursing staff and valuable human capital. Moreover, this research investigated mental workload and musculoskeletal disorders and their relationship with pediatric nurses’ intention to leave the service for the first time. Also, using full-census sampling method at the entire Bushehr Province was another strength of this research.

### Limitations

The present study faced some limitations that should be taken into account. Given that this was a cross-sectional study, it had limitations of cross-sectional studies and the obtained correlations should not be interpreted causally. Also, the research community was limited to nurses in Bushehr Province. Thus, generalizing the results to other regions requires conducting further research. Despite determining the sample size, some negative results may be due to the small sample size. Thus, conducting a study with a larger sample size is recommended. In this study, intention to leave service was investigated. More complete results could be obtained if nurses’ intention to leave the ward was also examined; though, it may be difficult to change wards as nurses have been trained for neonatal and pediatric wards. Moreover, investigating variables, especially musculoskeletal disorders, using questionnaires was among the limitations. It is suggested that longitudinal studies be conducted and laboratory data collection tools along with questionnaires such as musculoskeletal disorders be used in future studies. Finally, the researchers are recommended to design and test potential corrective interventions based on the prevalence and severity of musculoskeletal disorders in different organs as well as workload in the six mental workload domains.

## Conclusion

The results revealed no correlation between the mean total mental workload and intention to leave the service. However, among mental workload domains, intention to leave the service decreased with increasing the workload induced by effort. Musculoskeletal disorders were highly prevalent among nurses, but no correlation was found between musculoskeletal disorders and intention to leave the service among pediatric nurses. Also, increasing the number of night shifts increased intention to leave the service. Therefore, planning for appropriate allocation of night shifts, investigating the etiology and prevalence of musculoskeletal disorders and providing solutions for reducing mental workload should be among the main priorities of policymakers, while maintaining pediatric nurses’ motivation for making efforts.

## Data Availability

The datasets used and/or analyzed during the current study are available from the corresponding author upon reasonable request.

## Abbreviations

CMDQ: Cornell Musculoskeletal Discomfort Questionnaire
NASA‑TLX: National Aeronautics and Space Administration‑Task Load Index
NEXT: The Natural Experiments Study Group
